# Infrared Spectroscopic Study of Methane Ice, Pure and in Mixtures with Polar (H_2_O) and Nonpolar (N_2_) Molecules

**DOI:** 10.1021/acs.jpca.2c00287

**Published:** 2022-03-18

**Authors:** Shahnewaz M. Emtiaz, Francis Toriello, Jiao He, Gianfranco Vidali

**Affiliations:** † Physics Department, Syracuse University, Syracuse, New York 13244, United States; ‡ Max Planck Institute for Astronomy, Königstuhl 17, D-69117 Heidelberg, Germany

## Abstract

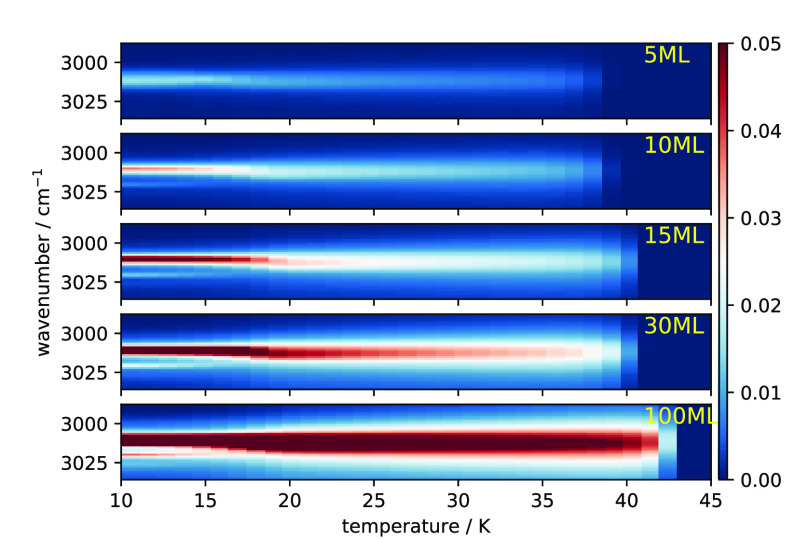

Mid-infrared studies of fundamental modes of ices of pure CH_4_ and its mixtures with polar (H_2_O) and nonpolar (e.g., N_2_) molecules are essential in order to learn the state of aggregation and thermal history of ices present in the interstellar medium and outer solar system bodies. Such data will be useful in the interpretation of observational data from the James Webb Space Telescope. Using an ultrahigh vacuum apparatus, we conducted reflection–absorption infrared spectroscopy measurements in the mid-IR range of pure methane ice and methane-containing ice mixtures of interest to interstellar and solar system ice chemistry, e.g., with H_2_O and N_2_ molecules. We found that nuclear spin conversion (NSC) in solid methane and its crystalline structures is affected—in different ways—by the presence of H_2_O and N_2_. Specifically, we found a relationship between the thickness and the solid-state ordering transformation in methane thin films. This new study of the NSC of pure CH_4_ ice and of the CH_4_:H_2_O ice mixture at 7 K is carried out in relation to the segregation of H_2_O using the ν_1_ and ν_2_ IR inactive modes of methane. The diffusion of N_2_ and CH_4_ in the CH_4_:N_2_ ice mixture with temperature cycling has been studied to obtain the relationship between IR features and the state of aggregation of the ice.

## Introduction

Solid methane and its mixtures with other volatiles are present in ocean beds as clathrate hydrates,^1^ in icy objects in the solar system,^2,3^ and in interstellar ices.^4−7^ In particular, methane ice has been detected in different solar system bodies either neat or mixed with other volatile molecules in different mixing ratios. Methane is found at the single digit percent level in interstellar ices^4^ but is abundant in outer solar system bodies, such as Titan, Triton, Pluto, and others. Its importance is related to the fact that is considered a key molecule in the development of prebiotic life.^8,9^

At cryogenic temperatures and low pressures, there are two recognized and well-documented solid phases.^10^ Phase I is the equilibrium phase at *T* > 20.4 K. It is a face-centered cubic (fcc) crystal with orientationally disordered CH_4_ molecules. Phase II is stable below 20.4 K and consists of an fcc lattice with eight ferrorientational sublattices (orientationally ordered sublattices, six with dihedral symmetry *D*_2*d*_ and two sublattices of hindered rotators with octahedral symmetry *O*_*h*_). Thus, in the primitive cell, the six CH_4_ that are orientationally ordered are subject to both the crystalline and orientational fields, while at the location of the two virtually free rotators, the octupolar orientational field vanishes.^11^ Recently, a metastable phase of solid methane at *T* < 7.0 K was discovered; it is a crystalline phase with orientational order between Phase II and Phase I.^12^

In the mid-IR, methane has two active vibrational modes, a C–H stretching at 3009 cm^–1^ (3.32 μm, ν_3_) and a deformation mode (ν_4_) at 1302 cm^–1^ (7.68 μm). In addition, methane solid goes through nuclear spin conversion (NSC) at cryogenic temperatures^12−15^ where the change in nuclear spin configuration influences the ro-vibrational spectrum. Molecular-level interaction between CH_4_ and H_2_O is accompanied by significant shifts in peak positions and spectral features in the near^16^ and mid-IR^17^ spectral regions. Specifically, the interaction of water with methane manifests itself in the detection of two additional modes, ν_1_ and ν_2_, which are inactive in pure solid methane.^18,19^

Methane has been detected in the interstellar medium (ISM)^20,21^ at low concentrations in water-dominated ices; when ejected in the gas phase it leads to a warm carbon-chain chemistry^22^ and the production of unsaturated hydrocarbons.^23^ In planetary systems, it is found in outer solar system bodies,^24^ in satellites of planets,^25^ and in water-rich comets.^26^ Near-IR features of methane ice on outer solar system bodies are blue-shifted,^27^ indicating that CH_4_ is mixed with N_2_, the other major component in those ices. The absence of combination modes (i.e., ν_1_ + ν_3_ and ν_2_ + ν_4_) involving IR inactive modes ν_1_ and ν_2_ in Triton’s spectrum has been used to infer that CH_4_ molecules are isolated and dispersed in N_2_:CH_4_ ice.^28−30^ A study of the shift positions and broadening of mid-IR spectral features of CH_4_ in CH_4_:H_2_O mixtures grown at 30 K is given in Gálvez et al. (2009).^31^ CH_4_:H_2_O mixtures in the 14–60 K range, and in particular the inactive ν_1_, are studied in Herrero et al. (2010).^32^ The possible origin of the activation of the ν_1_ mode of CH_4_ in CH_4_:H_2_O mixtures is investigated by means of solid-state calculations in Escribano et al. (2014).^33^

The goal of this work is to study the IR absorption features of pure methane ice and methane ice mixture with H_2_O and N_2_. We aim to quantify how the presence of these molecules in methane-rich ice produces changes in IR spectra. Furthermore, we want to see whether the state of aggregation can be discerned from IR features, as it was done for CO–CO_2_ ice mixtures^34^ and CO_2_ films on water ice.^35^ We proceeded as follows. We first measured the effect of deposition methods, thermal cycling, and deposition temperature on thin films of pure methane in ultrahigh vacuum (UHV) conditions. We then measured the spectral changes that methane ice experiences in the presence of H_2_O and N_2_. This allowed us to study the diffusion and segregation process in CH_4_:H_2_O and CH_4_:N_2_ ice mixtures.

## Experimental Methods

Experiments were performed at Syracuse University in an UHV chamber in which the pressure can routinely reach 4.5 × 10^–10^ Torr after a bake-out; see He et al.^36^ for details; here only the main features that are relevant to this study are summarized. Gases were deposited on a gold-coated copper disk mounted on a closed cycle cryocooler (ARS DE-204 4K). The temperature of the sample was monitored and manipulated by a calibrated silicon diode placed behind the sample and paired with a Lakeshore 336 temperature controller. A cartridge heater installed behind the sample can be used to heat the sample. By controlling the heating output of the cartridge heater, the temperature can be adjusted between 5 and 300 K with an accuracy of better than 50 mK.

Methane ices were grown on the sample disk by introducing methane through a stepper motor-controlled UHV variable leak valve. A LabVIEW program controlled the deposition rate and thickness. In all experiments, 100 monolayers (ML, defined as 10^15^ molecules/cm^2^) of ice were formed at a relatively high deposition rate of 100 ML/min. This deposition dose was calculated using the impingement rate; see the Appendix of He et al.^36^ for details. For the deposition of CH_4_ and H_2_O, two separate UHV variable leak valves were used. Distilled water underwent at least three freeze–pump–thaw cycles before being sent into the chamber. Premixed CH_4_ and N_2_ gas was deposited using a single leak valve for CH_4_:N_2_ mixture deposition except for one occasion: a molecular beamline was used to deposit 100 ML of CH_4_ for the experiment presented in Figure 4. The deposition of methane via the beamline was much slower, by a factor 100, with respect to the gas-phase deposition.

In most of the experiments, methane was deposited at 10 K, unless otherwise mentioned. A Nicolet 6700 FTIR in the reflection absorption infrared spectroscopy (RAIRS) configuration with an incident angle of ∼78° was used to obtain mid-IR spectra of the ice. Spectra were measured and averaged every 20 s at a resolution of 1 cm^–1^. To measure the NSC, the IR spectra were continuously monitored at a fixed temperature for different lengths of time, depending on the features being measured. For example, the isothermal experiments in which the ice was brought and kept at 7 K from the deposition temperature of 30 K lasted approximately 12 h. In other experiments, the ice was deposited at 10 K, kept at this temperature for several minutes, and then heated at 3 K/min to the desired temperature and subsequently cooled to 10 K for temperature-cycling experiments.Table 1 provides a synopsis of experiments performed for this work.

**Table 1:** List of Performed Experiments

no.	experiment summary	Figure
1	100 ML of CH_4_ deposited at temperatures between 7 and 30 K	1
2	Slow heating of CH_4_ ice between 2 and 100 ML and at temperature 10–45 K	2
3	Comparison of fast and slow deposited CH_4_ ice to investigate temporal change at 6 K	4
4	Comparison of a CH_4_:H_2_O mixture with variable concentrations deposited at 10 K	5
5	Effect of temperature cycling on CH_4_ IR modes for the CH_4_:H_2_O mixture	6
6	Comparison of a CH_4_:N_2_ mixture with variable concentrations deposited at 10 K	7
7	Effect of temperature cycling on CH4 IR modes for the CH_4_:N_2_ mixture	8

## Results and Analysis

We carried out two sets of experiments. In the first set, we studied the IR signatures of crystalline phases of methane under different conditions. For these experiments, pure methane ice of different thicknesses was deposited at either 6 or 10 K. Then methane ice was either kept at a specific temperature for an extended period of time or heated slowly at 3 K/min until the methane ice desorbed past 40 K. In the second set, methane ice mixed with either water or nitrogen was deposited at 10 K, and then the ice went through temperature cycling (heating and cooling) to study the state of the aggregation of the ice through changes in IR features during the process.

### Dependence of Methane Crystalline Phases on Deposition Temperature

**Figure 1:**
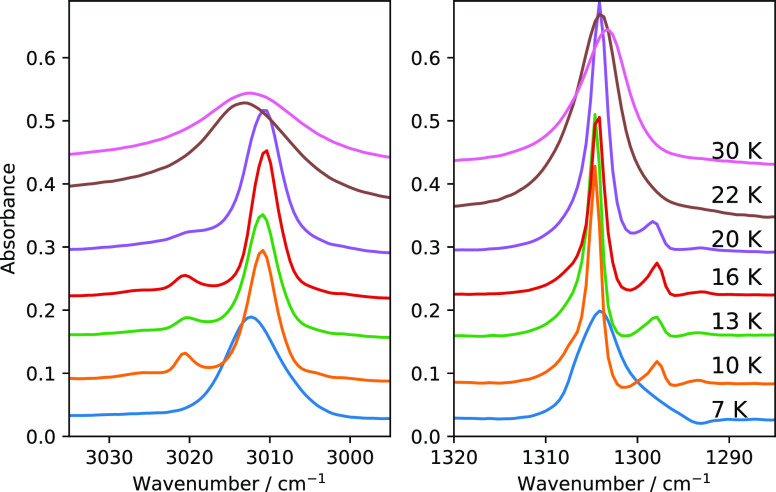
Mid-IR vibration modes of 100 ML of methane ice (ν_3_ (left) and ν_4_ (right)) deposited at 7, 10, 13, 16, 20, 22, and 30 K. The traces are displaced vertically for clarity.

Experiments to characterize the Phase I–Phase II transition in methane ice are typically conducted in closed cells.^11^ In such experiments, the transition happens abruptly at 20.4 K. However, from an analysis of methane thin films deposited in the 7–30 K range, we observe that the transition from Phase II to Phase I occurs over a temperature range. Figure 1 shows the ν_4_ and ν_3_ modes of pure methane ice deposited at different temperatures. At 7 K the ice is in Phase II*, which is a metastable phase without the presence of band splitting due to NSC.^12^ In pure methane ice, satellite peaks emerge near ν_4_ and ν_3_ due to IR-allowed transitions brought about by conversion of the nuclear spins of the hydrogen atoms in CH_4_ ice.^14^ For methane deposited at 10 K, the ice is in crystalline Phase II with the fully emerged band splittings due to NSC,^12,14^ in agreement with the results presented in a previous publication.^12^ As we raise the deposition temperature, we observe that the band splitting gradually diminishes due to the disruption of neighboring lattice sites. Between 20 and 22 K multiple peaks of ν_4_ and ν_3_ modes converge into a single broad peak for each mode due to orientational disordering at lattice sites. There is a significant blue shift from 3010.6 to 3014.2 cm^–1^ for the ν_3_ mode in that temperature range. Above 22 K, the ice forms an orientationally disordered fcc lattice.

### Effect of the Thickness of CH_4_ Thin Film on Phase II/Phase I Transition

**Figure 2:**
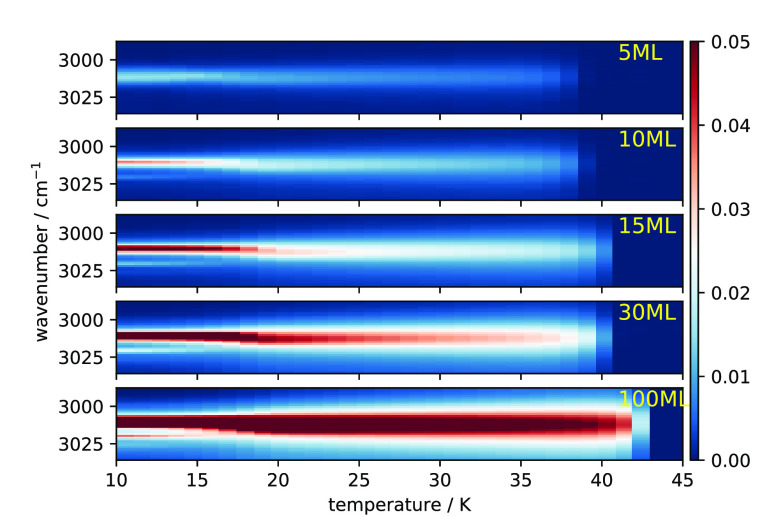
Intensity map of the ν_3_ mode during a slow heating (3 K/min) of the CH_4_ film with indicated thickness. The intensity scale is on the right.

**Figure 3:**
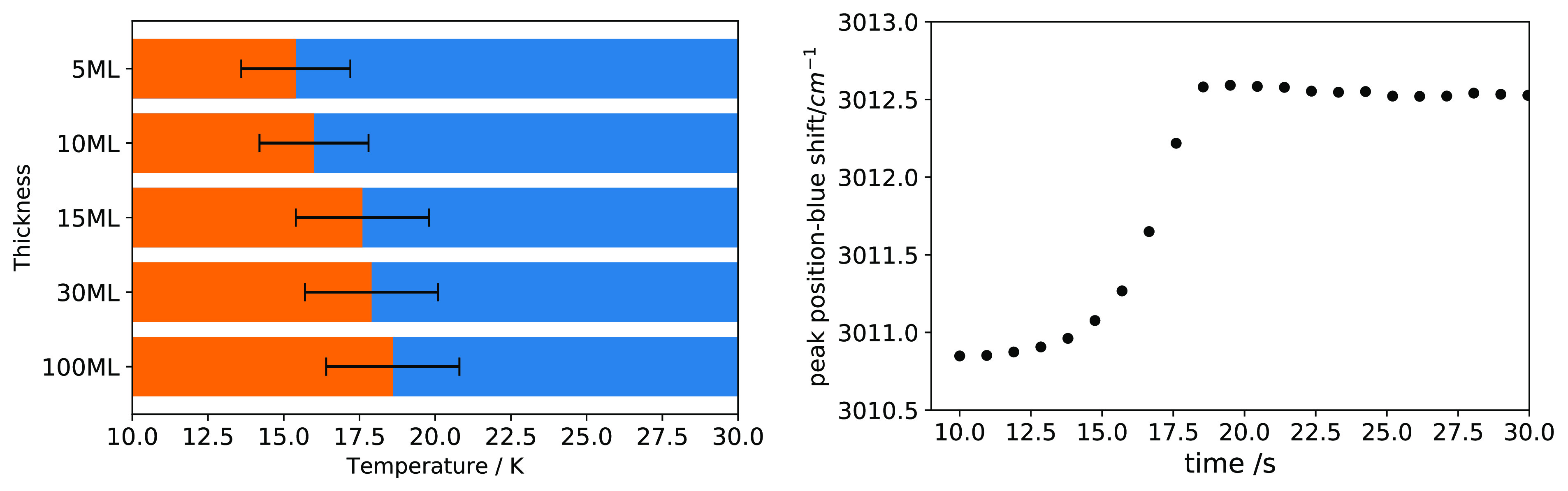
Temperature range where Phase II (left, orange) and Phase I (right, blue) are present for ices with the indicated thickness. The temperature range is obtained from Figure 2. Left panel: the horizontal black line represents the temperature range where a significant shift in the peak position of the ν_3_ mode occurs during slow heating up. Right panel: illustration of how the temperature range for the peak shift is determined, using the 15 ML measurement as an example. See text for details.

We deposited 5, 10, 15, 20, 30, and 100 ML of CH_4_ on a substrate at 10 K, and then heated the sample from 10 to 45 K at a rate of 0.1 K/s. The bending mode ν_3_ absorption spectra for all these thicknesses, normalized to the maximum absorption of all the spectra for all the spectra of the same thickness during the heating process, are shown in Figure 2. When solid methane goes through the orientational phase transition (from Phase II to Phase I), there is a change in the IR peak position and spectral features due to a change in the lattice field^14^ (for convenience’s sake, we call the phase transition an orientational phase transition irrespective if it goes from an orientational disordered to an orientationally ordered state or vice versa). We obtained the temperature range for the phase transition based on an analysis of the shifting of the peak position. The right panel of Figure 3 illustrates how the temperature range is determined, using the result of 15 ML as an example. Briefly, a Gaussian distribution is used to fit the ν_3_ peak, and the position is obtained for each spectrum during the warm-up. More details of the fitting scheme are described in Emtiaz et al.^12^ The peak position is then plotted as a function of temperature and is shown in the right panel of Figure 3. We take the temperature at which the peak position is shifted by at least 0.25 cm^–1^/K as the starting point of the phase transition. The end point of the phase transition is more obvious from the plot. The temperature range for the phase transition for methane ices of different thicknesses is then shown in the left panel of Figure 3. At 5 ML coverage the transition temperature is 16.2 K (Figure 3). In this case, the disruption of neighboring lattice field sites is accelerated due to the thinness of the film. On the other hand, for 15 ML and thicker ice the transition temperature is a little less than 20.4 K, the value for bulk CH_4_ ice. These experiments suggest that methane ice experiences thin-film effects for coverage below 15 ML. For higher coverage (≥100 ML), the ice is independent of thin-film effects.

### Dependence of NSC on the Deposition Rate of CH_4_

**Figure 4:**
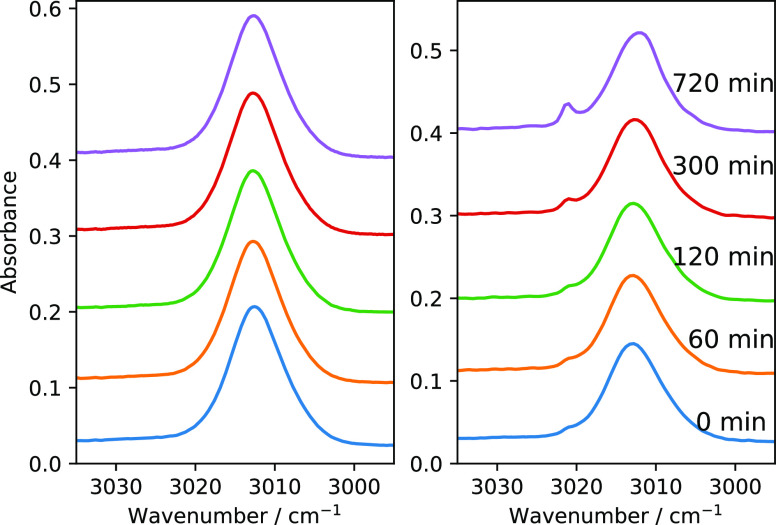
ν_3_ mode of 100 ML of methane solid deposited at 6 K. Left panel: temporal change of CH_4_ ice deposited through a UHV leak valve (deposition from background gas) at a rate of 100 ML/min. Right panel: same as in the left panel but for CH_4_ ice deposited through the molecular beamline at a rate of 1 ML/min.

We know that at 6 K the methane thin film is in a metastable phase; at that temperature nuclear spin conversion does not take place over laboratory times.^12^ As we increase the temperature above 7 K, we observe an exponential increase in the relaxation rate up to 8.5 K.^12^ Depending on the deposition rate, we found different rates of NSC for methane ice. The left panel of Figure 4 shows 100 ML of methane ice deposited at 6 K and kept for 12 h. Methane ice was deposited by filling the chamber background with methane gas through a UHV leak valve at a rate of 100 ML/min. In this background-deposited methane ice, we do not see any emergence of band splitting of the ν_3_ mode, which is a sign of NSC in IR spectroscopy. On the other hand, the right panel of Figure 4 shows 100 ML of methane ice deposited with the molecular beamline; the ice was kept at 6 K for a similar amount of time as for the background deposition. In this case, we observe the emergence of band splitting upon the completion of deposition. It takes 100 min to finish the deposition through the beamline compared to 1 min using the UHV leak valve. Initially (see bottom two traces on the right), we see the emergence of the R(0) band (3010.9 cm^–1^) for the ν_3_ mode and the Q(1) band (1297.8 cm^–1^) for the ν_4_ mode. As we keep the ice at 6 K for 12 h, we see that the band splitting is more pronounced. In the ice prepared slowly, orientational ordering at lattice sites takes place; therefore, we see a significant rate of NSC even at 6 K. This fact further strengthens our argument that at 6 K we observe a metastable phase of methane which is a crystalline phase with an orientational ordering between Phase I and Phase II.^12^

### Ice Mixtures of CH_4_ and H_2_O with Different Ratios

**Figure 5:**
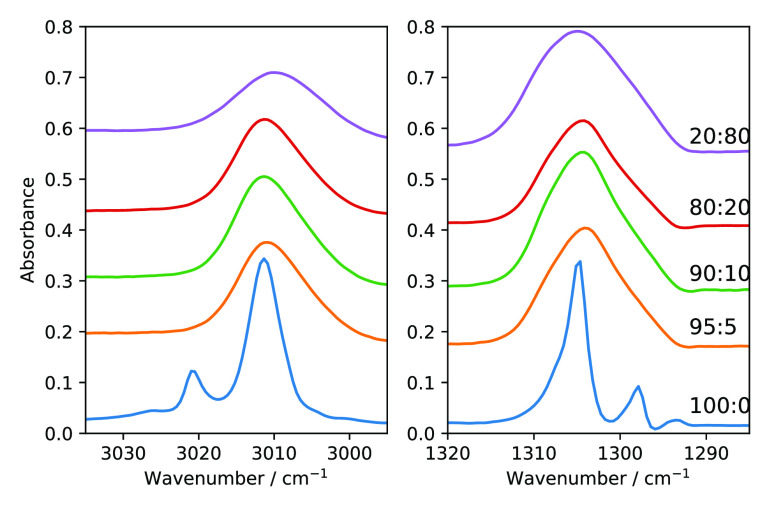
ν_3_ (left) and ν_4_ (right) modes for a CH_4_:H_2_O mixture with different concentrations. All the mixtures were deposited at 10 K.

Figure 5 shows IR data of ν_4_ and ν_3_ modes of pure methane and methane–water mixture with different mixing ratios. We observe a significant change in IR features and fwhm values for increasing amounts of water mixed with methane. For CH_4_:H_2_O (=95:5) we observe that the peak position is red-shifted by 0.8 and 0.7 cm^–1^ for ν_4_ and ν_3_, respectively. The fwhm becomes 14 and 7 cm^–1^ for the ν_4_ and ν_3_ modes, respectively, which is a significant increase from pure methane. The red shift in the peak position is more significant for the ν_3_ than for the ν_4_ mode. All the relevant values are listed in Table 2.

**Table 2:** ν_4_ and ν_3_ Band Positions, Shifts, and fwhm of CH_4_:H_2_O with Different Mixing Ratios

mode	mixing ratio	peak position (cm^–1^)	fwhm (cm^–1^)	shift (cm^–1^)
ν_4_	100:0	1304.8	4.5	
	95:5	1304.0	14	–0.8
	90:10	1303.9	14.5	–0.9
	80:20	1303.9	15.5	–0.9
	20:80	1304.2	21	–0.6
ν_3_	100:0	3010.9	7	
	95:5	3010.2	10.5	–0.7
	90:10	3010.0	11.0	–0.9
	80:20	3009.9	11.2	–1.0
	20:80	3008.1	14.0	–2.8

### Segregation of Water in a CH_4_:H_2_O Ice Matrix

**Figure 6:**
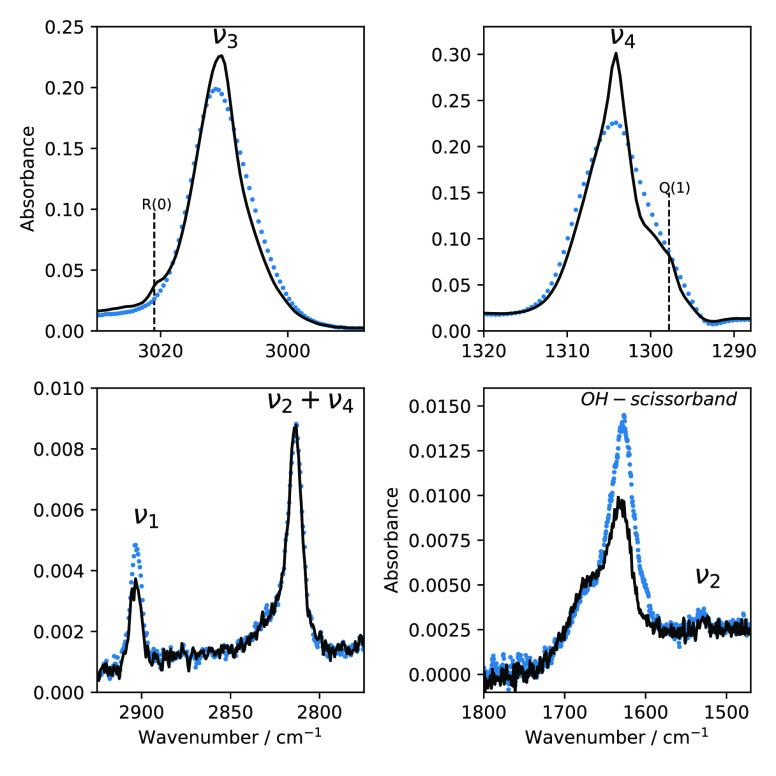
Effect of thermal cycling in a CH_4_:H_2_O (=80:20) mixture. The blue dotted IR trace shows IR spectra of ice deposited at 10 K, while the solid black line is for ice after it has undergone one round of heat cycling (from 10 to 30 K and back down to 10 K).

In this section we investigate the CH_4_:H_2_O ice using the CH_4_ IR inactive modes. Pure methane has two IR inactive modes: ν_1_ (2904.5 cm^–1^) and ν_2_ (1540.0 cm^–1^). When water is mixed with methane, there is no band splitting due to NSC in Phase II CH_4_:H_2_O mixtures at 10 K. However, the presence of water activates the IR inactive modes.^18,19^Figure 6 shows IR spectra of CH_4_:H_2_O at two different stages of the same experiment. The dotted marked spectra represent CH_4_:H_2_O after the deposition at 10 K. The solid line shows the band after the ice is taken to 30 K and then cooled back to 10 K. Two intense bands are observed at 3500 cm^–1^ (not shown) and at 1635 cm^–1^, caused by O–H stretching and O–H–O scissors-bending,^37^ respectively. We observe significant changes in ν_4_ and ν_3_ modes before and after temperature cycling. Band splitting such as R(0) for ν_3_ and Q(1) for ν_4_ emerges after temperature cycling. Band strengths of ν_1_ and of O–H–O scissors-bending decrease by about 50% after the heating and cooling process. This change in IR features indicates that fewer CH_4_ molecules are in contact with water molecules; therefore, a partial segregation of water in the CH_4_:H_2_O ice has taken place. This segregation process happens during orientational reordering of lattice field sites of methane molecules during temperature cycling.

Another piece of evidence of segregation is as follows. Nuclear spin conversion occurs in methane ice between 7 and 11 K.^12^ There is no NSC for CH_4_:H_2_O ice deposited at 10 K and kept at that temperature for 30 min. However, if the CH_4_:H_2_O ice is taken to 30 K and then rapidly cooled to 7 K and kept for 100 min, NSC splitting is observed, as in the case of pure methane ice. The fact that the CH_4_:H_2_O ice mixture—when annealed at 30 K—goes through the NSC process similar to pure methane ice (while the mixture quenched to 7 K does not) indicates that water segregated and left patches of pure methane where NSC can take place. A similar segregation process has been observed in CO:CO_2_ = 9:1 ice mixtures when CO undergoes a phase transition from amorphous to crystalline.^34^ During the transition, CO_2_ molecules form clusters. The segregation of H_2_O from the CH_4_:H_2_O mixture during NSC may be a process that is common on icy bodies in the solar system. All the relevant values of ν_3_ and ν_4_ vibrational modes peak position and band shift are listed in Table 3.

**Table 3:** Band Positions and Shifts of Pure Methane and CH_4_:H_2_O = 95:5 During NSC

mode	band assignment	pure methane (cm ^**–**1^)	CH_4_:H_2_O (cm ^–1^)	shift (cm ^–1^)
ν_4_	R(0)	1307.3		
	R(1)	1301.2		
	Q(1)	1297.8	1298.7	0.9
	P(1)	1294.4		
	P(2)	1293.2	1294.4	1.2
ν_3_	R(0)	3021.0	3020.1	–0.9
	R(1)	3026.6	3027.2	0.6
	Q(1)	3010.9	3011.4	0.5
	P(1)	3004.1		
	P(2)	3000.6		

### Ice Mixtures of CH_4_ and N_2_ with Different Ratios

**Figure 7:**
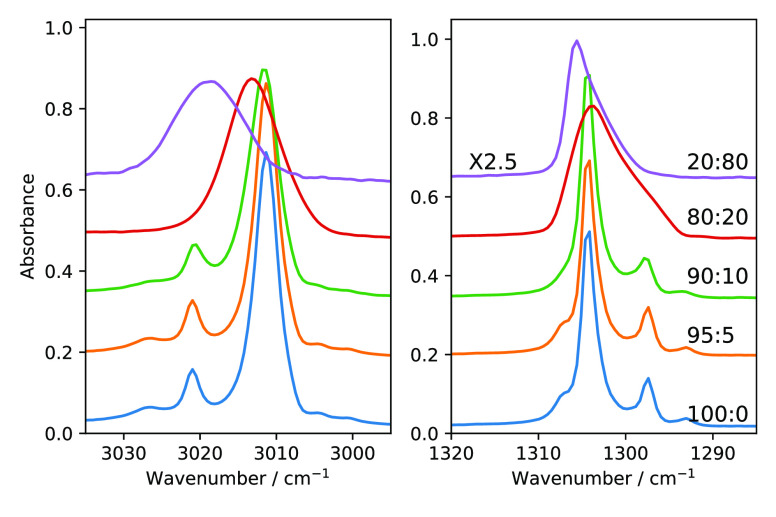
RAIRS spectra of solid methane in the ν_3_ and ν_4_ vibrational modes of the CH_4_:N_2_ mixture for various concentrations. All the mixtures were deposited at 10 K.

N_2_ is a nonpolar molecule, and the unit cell of N_2_ has a lattice parameter of 5.64 Å,^38^ which is close to the value of the lattice parameter of fcc methane solid, 5.89 Å. Thus, it is a reasonable assumption that in the CH_4_:N_2_ mixture, N_2_ is in a substitutional site rather than in an interstitial site as the ices have similar lattice parameters and both have an fcc structure. Figure 7 shows the IR data of CH_4_ ν_4_ and ν_3_ modes of CH_4_:N_2_ with different mixing ratios. We observe that there is little change once we introduce a small amount of N_2_ in the ice matrix. The CH_4_ lattice symmetry is disrupted once the impurity level reaches 20%. At the CH_4_:N_2_ = 80:20 ratio the satellite peaks of CH_4_ ν_4_ and ν_3_ modes completely disappear which indicates that the N_2_ molecules in the ice matrix cause enough disruption of the symmetry of the original CH_4_ ice that the collective behavior giving rise to NSC is suppressed. We see a significant change in IR features and FWHM values in CH_4_:N_2_ = 80:20. In this case, the peak position is red-shifted by 0.4 and 2.3 cm^–1^ for ν_4_ and ν_3_, respectively. The fwhm becomes 9.4 cm^–1^ for ν_4_ and 9.5 cm^–1^ for ν_3_ modes, which is a big increase from pure methane ν_4_ and ν_3_ modes, 4.1 and 6.3 cm^–1^, respectively. The blue shift in the peak position is more significant in the ν_3_ mode than in the ν_4_ mode. All the relevant values are listed in Table 4.

**Table 4:** Band Positions, fwhm, and Shifts of ν_4_ and ν_3_ Modes of CH_4_:N_2_ for Various Mixing Ratios

mode	mixing ratio	peak position (cm^–1^)	fwhm (cm^–1^)	shift (cm^–1^)
ν_4_	100:0	1304.8	4.5	
	95:5	1304.9	4.7	0.1
	90:10	1305.0	4.8	0.2
	80:20	1304.6	9.4	0.4
	20:80	1306.4	6.5	1.4
ν_3_	100:0	3010.9	7	
	95:5	3011.0	7.2	0.1
	90:10	3011.2	7.5	0.3
	80:20	3013.2	9.5	2.3
	20:80	3018.6	12.8	8.5

**Figure 8:**
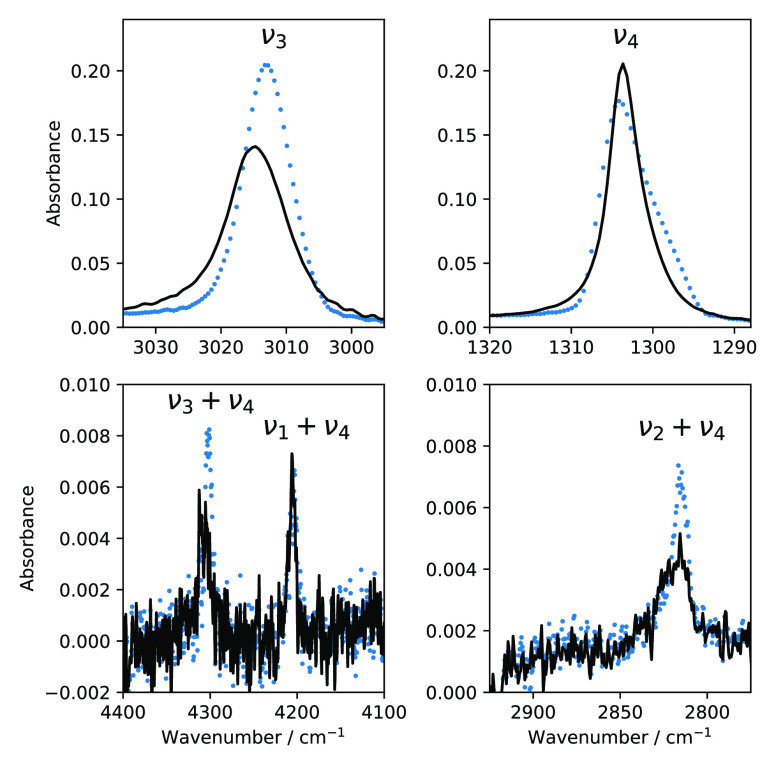
Effect of temperature cycling (from 10 to 20 K and back to 10 K) on the IR ν_3_ (top-left), ν_4_ (top-right), ν_3_ + ν_4_ (bottom-left), and ν_2_ + ν_4_ (bottom-right) bands in a CH_4_:N_2_ (=80:20) mixture. The blue dotted line shape represents the ice mixture upon deposition, and the solid black line represents the ice that is taken to 20 K and then cooled back to 10 K.

Figure 8 shows IR spectra of CH_4_:N_2_ (=80:20) before and after thermal cycling from 10 to 20 K and back to 10 K. It suggests that there is a structural rearrangement in the ice mixture as the ice mixture undergoes a heat cycling between 10 and 20 K. We observe a significant change in the ν_3_ mode and ν_2_ + ν_4_ and ν_3_ + ν_4_ combinations modes. The peak position of the ν_3_ mode blue-shifts 3012.5 cm^–1^ to 3016.3 cm^–1^ , and the fwhm becomes 14.8 cm^–1^ from 9.5 cm^–1^. In the case of combination modes, ν_2_ + ν_4_ loses its band strength by 50% and ν_3_ + ν_4_ blue-shifts. The broadening of the bands after the thermal cycling suggests that there has been a loss of symmetry or ordering. A detailed study of the CH_4_/N_2_ diffusion process will be presented in a forthcoming work.

### Application to Observations

As mentioned in the Introduction and in the references cited therein, methane in the solid state has been found in many space environments, such as the interstellar medium, outer planets, solar system objects, and comets. The results related to NSC in solid methane apply to those interstellar environments where the temperature is significantly lower than 10 K. Given the low concentration of CH_4_ in the solid state in interstellar environments, the formation of CH_4_ aggregates likely requires thermal input for the diffusion of CH_4_ molecules, and we showed that IR features can be used to characterize the thermal history of the mixture. In planetary environments, including comets, the interaction of methane with water is important, as well the detection of IR features that are affected by thermal cycling. Our results show how thermal cycling of a CH_4_:H_2_O ice mixture induces the segregation of water and methane and how this change can be followed by the analysis of IR features.

## Summary

The main findings of our measurements of mid-IR bands of pure methane ice and in mixtures with water and nitrogen are as follows:

- The orientational ordering transition (Phase II to Phase I) in methane ice films occurs over a range of temperature in contrast with measurements of bulk methane ice in closed cell experiments where the transition is abrupt.^39^
- Nuclear spin conversion at cryogenic temperatures depends on the deposition rate. NSC is virtually absent in a fast deposition from background gas (100 ML/min), but it shows up in a slow deposition of ice at 6 K (1 ML/min) from the beamline.
- A small (5*%*) amount of water present in the ice matrix causes changes in CH_4_ IR bands. The peak position of the CH_4_:H_2_O matrix is red-shifted as we increase the amount of water in the mixture. Similarly, the fwhm of the CH_4_:H_2_O broadens with the increased percentage of water.
- The introduction of an amount of water greater than 5% during deposition at low temperatures (10 K) suppresses NSC, and forbidden CH_4_ bands (ν_1_) appear. When the ice is taken to 30 K and then cooled back to 10 K, these forbidden bands get greatly reduced (50% for a CH_4_:H_4_O = 80:20 mixture), indicating that there are fewer CH_4_ molecules in contact with H_2_O molecules. This is an indication that water is partially segregated from CH_4_.
- Compared to CH_4_:H_2_O mixtures, in CH_4_:N_2_ mixtures a much larger amount (20%) of N_2_ is required to suppress NSC. Once the N_2_ fraction is over 20%, there is a blue shift of peak positions of ν_3_ and ν_4_ modes. As the temperature is raised, the orientational symmetry of the CH_4_ ice structure changes.

Taken all together, these observations should help the interpretation of observations of CH_4_ ice in interstellar and planetary environments, such as its thermal history and its degree of mixing with water and nitrogen molecules.
